# The LonDownS adult cognitive assessment to study cognitive abilities and decline in Down syndrome

**DOI:** 10.12688/wellcomeopenres.9961.1

**Published:** 2016-11-15

**Authors:** Carla M. Startin, Sarah Hamburg, Rosalyn Hithersay, Amy Davies, Erin Rodger, Nidhi Aggarwal, Tamara Al-Janabi, André Strydom

**Affiliations:** 1 UCL Division of Psychiatry, University College London, London, UK; 2 The LonDownS Consortium, University College London, London, UK; 3 Faculty of Health and Medical Sciences, University of Surrey, Guilford, UK

**Keywords:** Down syndrome, intellectual disability, Alzheimer’s disease, dementia, cognition, memory, executive function, motor coordination

## Abstract

Background: Down syndrome (DS), the most common genetic cause of intellectual disability, is associated with an ultra-high risk of developing Alzheimer’s disease. However, there is individual variability in the onset of clinical dementia and in baseline cognitive abilities prior to decline, particularly in memory, executive functioning, and motor coordination. The LonDownS Consortium aims to determine risk and protective factors for the development of dementia and factors relating to cognitive abilities in people with DS. Here we describe our cognitive test battery and related informant measures along with reporting data from our baseline cognitive and informant assessments.

Methods: We developed a cognitive test battery to assess general abilities, memory, executive function, and motor coordination abilities in adults with DS, with informant ratings of similar domains also collected, designed to allow for data on a broad range of participants. Participants (n=305) had a range of ages and abilities, and included adults with and without a clinical diagnosis of dementia.

Results: Results suggest the battery is suitable for the majority of adults with DS, although approximately half the adults with dementia were unable to undertake any cognitive task. Many test outcomes showed a range of scores with low floor and ceiling effects. Non-verbal age-adjusted IQ scores had lower floor effects than verbal IQ scores. Before the onset of any cognitive decline, females aged 16-35 showed better verbal abilities compared to males. We also identified clusters of cognitive test scores within our battery related to visuospatial memory, motor coordination, language abilities, and processing speed / sustained attention.

Conclusions: Our further studies will use baseline and longitudinal assessments to explore factors influencing cognitive abilities and cognitive decline related to ageing and onset of dementia in adults with DS.

## Introduction

Down syndrome (DS) is the most common genetic cause of intellectual disability (ID) and is caused by the presence of an additional chromosome 21. DS has a UK incidence of approximately 1 in 1000 live births ( Wu & Morris, 2013). The life expectancy for individuals with DS has risen dramatically over the previous 50 years; a recent study estimated current life expectancy to be almost 60 ( Englund *et al.*, 2013). With this increase in life expectancy it has become apparent DS is associated with an ultra-high risk of developing Alzheimer’s disease (AD) compared to typically developing individuals ( Wiseman *et al.*, 2015). A recent study estimated lifetime risk for dementia based on cumulative incidence may be as high as 95.7% by age 68, with an age-related increase from 26.1% at age 50 ( McCarron *et al.*, 2014).

This increased risk of dementia is thought to be largely due to the overexpression of genes on chromosome 21. Of particular interest is the amyloid precursor protein ( *APP*) gene, mutations in which have been associated with early onset AD in the typically developing population. Deposits of amyloid, a characteristic feature of AD and encoded by the *APP* gene, are reported to be present in the brains of almost all adults with DS with full trisomy 21 over the age of 30 ( Mann, 1988; Wisniewski *et al.*, 1985). Despite this, there is considerable variability in the clinical presentation and age of onset of dementia in DS; some adults receive a dementia diagnosis before age 40 while others do not show signs of dementia until they reach their 60s, with a mean age of diagnosis of 55 ( Coppus *et al.*, 2006; Holland *et al.*, 1998; Margallo-Lana *et al.*, 2007; McCarron *et al.*, 2014; Tyrrell *et al.*, 2001). This wide variability suggests there are a number of risk factors for the development of clinical dementia in addition to *APP* overexpression, as well as protective factors against its development.

Dementia in DS develops on a background of an altered cognitive profile. Later developing brain networks, including the prefrontal cortex (PFC), hippocampus, and cerebellum, have been suggested to be most affected in DS ( Edgin, 2013). Structural MRI studies have reported smaller brain volumes in these regions in DS before the onset of AD ( Aylward *et al.*, 1999; Beacher *et al.*, 2010; Carducci *et al.*, 2013; Pinter *et al.*, 2001a; Pinter *et al.*, 2001b; Teipel *et al.*, 2003), and delayed hippocampal myelination has been demonstrated ( Ábráham *et al.*, 2012). In addition, altered frontal functional connectivity ( Anderson *et al.*, 2013; Pujol *et al.*, 2015) and white matter integrity ( Powell *et al.*, 2014) have been reported in DS. Those with dementia show further reduction in hippocampal volumes ( Aylward *et al.*, 1999; Beacher *et al.*, 2009) and decreased frontal white matter integrity ( Powell *et al.*, 2014) compared to those without dementia.

Altered development of the PFC, hippocampus and cerebellum in DS is supported by studies reporting related cognitive impairments, specifically in executive function, memory and motor coordination respectively. Individuals with DS show impaired executive functioning abilities compared to both mental age (MA) matched typically developing controls and individuals with non-DS ID ( Lanfranchi *et al.*, 2010; Rowe *et al.*, 2006), although one aspect of executive functioning, working memory, has been reported not to be affected in DS compared to MA controls ( Pennington *et al.*, 2003). Both verbal and visuospatial memory have been reported to be impaired in DS compared to MA controls ( Pennington *et al.*, 2003), in particular as memory load increases ( Visu-Petra *et al.*, 2007). It has further been suggested individuals with DS show relatively poorer verbal compared to visuospatial memory ( Baddeley & Jarrold, 2007; Jarrold *et al.*, 2002; Lanfranchi *et al.*, 2012), and visual object memory is more impaired than visual spatial memory ( Vicari *et al.*, 2005). Finally, individuals with DS have been reported to show slower motor responses compared to MA controls ( Edgin *et al.*, 2010; Frith & Frith, 1974). Although these general profiles of cognitive abilities are found for individuals with DS at the group level, there is a large variability both across and within individuals in cognitive profiles.

This cognitive profile in DS has been proposed to affect the presentation of dementia symptoms. Decline in frontal function ( Holland *et al.*, 1998; Holland *et al.*, 2000), characterised by executive function impairments ( Adams & Oliver, 2010; Ball *et al.*, 2008) and behavioural and personality changes ( Ball *et al.*, 2006; Dekker *et al.*, 2015), has been implicated as an early dementia-related change in DS. Memory impairments, usually associated with AD in the general population, are also found in adults with DS and dementia ( Ball *et al.*, 2006; Kittler *et al.*, 2006), with changes in praxis occurring later ( Dalton *et al.*, 1999).

### Concept and aims

The London Down Syndrome Consortium (LonDownS) aims to identify risk and protective factors for the development of the clinical signs of dementia in DS. This will inform understanding of the development of AD and identify potential mechanisms as well as predictive phenotypes. We also aim to establish the pre-dementia cognitive profile of adults with DS, allowing us to identify factors relating to cognitive abilities. This will help to inform interventions to influence developmental trajectories across the lifespan.

Our study therefore requires detailed cognitive assessments that allow for data on the broadest range of participants possible in terms of age and abilities, with minimal floor and ceiling effects. We also took into account the typical cognitive difficulties in this population, such as expressive language impairment, as well as co-morbidities such as hearing and vision problems. We therefore compiled a cognitive assessment battery requiring minimal verbal responses and using informant ratings of similar domains.

Here, we describe the LonDownS cognitive test battery for adults with DS, and provide data on baseline cognitive and related informant assessments.

## Methods

### Participants

***Cohort 1: adults aged 36 years and over.*** We have recruited and completed baseline assessments for 181 adults aged 36 years and over, with (n=51) and without (n=130) a clinical diagnosis of dementia, with longitudinal assessments planned to assess cognitive decline. Longitudinal assessments are essential to assess cognitive decline in individuals with DS due to the presence of an ID potentially confounding test results. Two additional adults were assessed then excluded from analyses after genetic testing revealed no additional chromosome 21, mosaicism or translocation. One further adult withdrew after starting the initial assessment.

***Cohort 2: adults aged 16–35 years.*** We have recruited and assessed 124 adults aged 16–35 years. These adults have initially been assessed once, to explore cross-sectional cognitive profiles of individuals with DS before the onset of dementia.

***Recruitment.*** Participants were recruited across England and Wales (focusing on the Greater London area and South East England) via local care homes, DS support groups and existing participant databases. We also established a network of National Health Service (NHS) Trust sites to identify and approach potential participants. Participants were given a gift voucher as compensation for their time, and we reimbursed all travel expenses.

***Inclusion and exclusion criteria.*** All participants were required to have a clinical diagnosis of DS. This was confirmed genetically using saliva or blood samples. We excluded participants with an acute physical or mental health condition, although when such participants recovered they were eligible for the study.

***Ethical approval and consent.*** Ethical approval was obtained for the LonDownS study from the North West Wales Research Ethics Committee (13/WA/0194). Participants with and without the capacity to consent were able to participate. Where individuals had capacity to consent we obtained written informed consent. Where individuals did not have capacity to consent a consultee was appointed and asked to sign a form to indicate their decision regarding the individual’s inclusion based on their knowledge of the individual and his/her wishes, in accordance with the UK Mental Capacity Act 2005.

### Assessment battery

Our battery was based on several established and novel assessments relevant to the cognitive profile and development of dementia in DS, including the Arizona Cognitive Test Battery (ACTB) ( Edgin *et al.*, 2010) which includes several computer tasks from the Cambridge Neuropsychological Test Automated Battery (CANTAB) ( CANTAB®, 2016). The ACTB was developed to assess a range of skills relevant to those brain areas most affected in DS, to have variable scores with low floor effects that are suitable for a range of ages and contexts, to be suitable for a non-verbal population, and to show good test-retest reliability. This battery was validated using individuals with DS aged 7–38. However, our previous pilot work showed some components of the ACTB had significant floor effects in older adults aged 45+ with DS, and some tests forming part of the battery were not able to distinguish between those with and without dementia ( Sinai *et al.*, 2016).

We therefore made several modifications to the ACTB. We excluded some tests for older adults (Cohort 1) based on our pilot results, specifically the virtual generated arena, cats and frogs, and finger sequencing. We added comparable table-top tests as our previous studies have supported their use in people with DS and found lower floor effects compared to computer tasks ( Sinai *et al.*, 2016). We also added informant-rated tools to cover similar cognitive domains as the neuropsychological test battery, allowing us to collect data on those unable to engage in cognitive testing.

A summary table of assessments can be found in the Supplementary material S1.

***Test administration.*** To avoid excessive burden to participants who were unable to engage in formal assessment and follow simple instructions (e.g. those with more severe dementia) the battery was only administered to those who were able to understand, meet thresholds for, and respond to the Kay vision test ( Kay, 1983), the Whisper hearing test ( Prescott *et al.*, 1999) and the first question of the KBIT-2 ( Kaufman & Kaufman, 2004). Adults who did not meet these thresholds did not complete any further tests in the battery, though their carers completed all informant questionnaires. In addition, we used the motor screening task (MOT) from the CANTAB ( CANTAB®, 2016) to familiarise participants with using the touchscreen. For this participants were required to press a cross on the screen at different locations for 10 trials.

Task order was counter-balanced across participants (see Supplementary material S2). We used a fixed order, but took a pragmatic approach that allowed flexibility where necessary. The assessment was completed in one session where possible, approximately 3 hours in duration, with a 10 minute break in the middle and additional breaks as necessary. Assessments took place where convenient for participants, usually in their homes, and occasionally using our testing rooms. Notes about the participant’s attention, co-operation, affect, and anxiety were made where appropriate throughout the assessment, including reasons for non-completion of tasks.

### Vision and hearing assessment

***Kay vision test.*** Participants’ visual acuity, wearing correction if appropriate, was tested using the Kay vision test ( Kay, 1983). Participants were asked to identify increasingly small pictures from 3m away, verbally or by pointing to the screening card, and the smallest size the participant could see was recorded. A threshold of 3/19 was used to identify those with significant vision problems that would invalidate cognitive test results. Only participants who met this threshold were administered further cognitive tasks.

***Whisper hearing test.*** Participants’ hearing abilities, using correction if appropriate, were tested using the Whisper test ( Prescott *et al.*, 1999), adapted for individuals with ID. The researcher stood behind the participant, 50cm from the midpoint between the ears on the top of the head, and whispered the name of one of eight objects (toothbrush, popcorn, ice cream, snowman, reindeer, hotdog, football, seesaw) displayed on the participant’s test card. Words were simple spondee words, i.e. contained two syllables with equal stress on each. The participant was asked to repeat the word or point to the correct picture. If the participant was unable to hear a whispered word a conversational, then loud voice, was used. The quietest level heard was recorded. Only participants who were able to hear and respond correctly to at least a loud voice were administered further cognitive tasks.

### Test of general abilities

***KBIT-2.*** We assessed general cognitive abilities using the Kaufman Brief Intelligence Test 2 (KBIT-2) ( Kaufman & Kaufman, 2004). The KBIT-2 consists of three subtests, two of which assess verbal IQ (verbal knowledge and riddles) and one assessing non-verbal IQ (matrices). Each subtest was started at item 1, and stopped after 4 consecutive incorrect answers. The KBIT-2 provides raw scores or age-dependent IQ scores. As we expected significant floor effects for IQ scores (i.e. an IQ of 40), we used raw scores as the main measure of general ability.

### Tests of memory

***CANTAB – PAL.*** The paired associates learning (PAL) task is a measure of visuospatial short-term memory from the CANTAB ( CANTAB®, 2016). Participants were required to remember locations of an increasing number of patterns in progressive stages, hidden behind boxes on the screen. If a particular stage was not completed in a maximum of 10 attempts the test terminated. The main outcome from this test was the first trial memory score: the number of pattern locations correctly remembered on the first trial for each stage attempted. The secondary outcome was the number of stages completed.

***CAMCOG – delayed incidental memory, verbal fluency and orientation.*** The Cambridge Cognitive Examination (CAMCOG) is a series of neuropsychological tests from the Cambridge Mental Disorders of the Elderly Examination (CAMDEX), used to assess cognitive impairments associated with dementia ( Roth *et al.*, 1986), and adapted to assess cognitive abilities in people with DS ( Hon *et al.*, 1999). The three tests used in our battery assess short-term memory (delayed incidental memory), frontal function (semantic verbal fluency) and participants’ knowledge of when it is (i.e. the day, month and year) and where they are (orientation).

Firstly, participants were administered the picture naming task, in which they were shown 6 pictures of objects and asked to name them. There were then two distractor tasks before incidental memory was tested: the verbal fluency task (see under tests of executive function) and the orientation task, in which participants were asked their full name, the day of the week, the month, the year, where they are, and the nearest city/town. For the orientation task the outcome is calculated from the number of questions answered correctly, with fewer points given if a clue was required. Finally, the delayed incidental memory task required participants to freely recall the pictures they saw earlier, then recognise them from 3 options. The outcomes for the incidental memory task were the number of objects correctly recalled and recognised.

***Delayed object memory.*** This test is a measure of short-term memory, based on the Fuld object memory test ( Fuld, 1980). We adapted this task to use 7 objects (toothbrush, comb, spoon, pencil, watch, coin and key) rather than 10 to reduce the memory load for participants. We also added a delayed memory trial (5 minute delay) in addition to two immediate memory trials to assess delayed as well as immediate memory. At the start of each trial participants named all seven objects and were instructed to remember them; any objects not correctly identified were named by the examiner. Participants’ memory was tested during two immediate recall trials followed by one 5-minute delayed recall trial. Immediately following each recall trial any objects not remembered were shown to the participant. During the delay wherever possible we collected physical measurements (height, weight, abdominal/head/neck circumference, gait, blood pressure, and pulse) from the participant. The outcome measures were the total number of objects correctly remembered in the two immediate memory trials combined and in the delayed memory trial.

***NAID – memory for sentences.*** This test of verbal memory is taken from the Neuropsychological Assessment of Dementia in Adults with Intellectual Disabilities (NAID) ( Oliver *et al.*, 1998). At baseline this test was administered to Cohort 2 only. Participants were asked to repeat 6 sentences after the researcher. The outcome measure was the number of words correctly remembered.

***ACTB – virtual generated arena.*** The virtual generated arena is a measure of visuospatial short-term memory, taken from the ACTB ( Edgin *et al.*, 2010). This task was adapted from the C–G arena ( Thomas *et al.*, 2001) and is based on the Morris water maze from the animal literature ( Morris, 1984). The arena task was only administered to Cohort 2. This task required participants to learn and remember where a hidden carpet was in a virtual room, using visual cues around the room. The main outcome was the percentage of time searching in the correct quadrant in the final test trial when no carpet is present.

### Tests of executive function

***CANTAB – IED.*** The intra/extra dimensional set shift (IED) task is a measure of rule learning and set shifting from the CANTAB ( CANTAB®, 2016). Participants were required to learn rules about which was the ‘correct’ of two presented patterns. When a rule was established (6 consecutive correct answers) there was a rule change and participants were required to learn a new rule in the next stage. If a particular stage was not complete (i.e. that rule was not ‘learnt’) in a maximum of 50 trials the task terminated. The two main outcome measures were the number of stages completed (measure of set shifting) and the number of stage 1 errors (measure of rule learning). Completing stages 2–7 required an intra-dimensional shift, completing stages 8–9 required an extra-dimensional shift (stage 1 required rule learning only with no shift).

***CANTAB – SRT.*** The simple reaction time (SRT) task from the CANTAB was originally proposed as a measure of attention ( CANTAB®, 2016), and was included in the ACTB as a measure of motor abilities ( Edgin *et al.*, 2010). Participants were required to press a button as soon as a white square appeared on the computer screen. There was an initial practice block of 24 trials, followed by two test blocks of 50 trials each. Outcome measures of interest were the standard deviation of the response time, which allows an estimate of consistency in response time and thus reflects attention levels during the task, the total number of correct responses, and mean response time.

***Semantic verbal fluency.*** Verbal fluency is a measure of frontal function ( Elfgren & Risberg, 1998). Participants were asked to name as many animals as they could in 1 minute. The main outcome was the number of unique animals named (including age and sex variations). The number of animals repeated and the total number of repetitions are outcomes of future interest.

***Tower of London.*** The Tower of London is intended to assess working memory and planning ( Shallice, 1982). Participants were required to move beads on a board to match presented configurations. We used a modified version of this task ( Strydom *et al.*, 2007), consisting of problems 1 to 5 from Krikorian *et al.* (1994) which can be completed in a minimum of 2–4 moves. Before commencing, the participant’s ability to name the colour of each bead was tested to ensure they could distinguish between them (e.g. they were not red-green colour blind). The outcome measure was calculated from the number of trials completed, with 2 points for trials completed in the minimum number of moves and 1 point for trials completed with more moves.

***ACTB – cats and frogs.*** The cats and frogs test measures rule learning and switching, inhibitory control, and working memory ( Edgin *et al.*, 2010) and is based on the Dots test ( Davidson *et al.*, 2006). We only administered this test to Cohort 2. Participants were required to learn two different rules in Stages 1 and 2 (the ‘cat’ and ‘frog’ rules respectively), and then combine them in Stage 3. For the ‘cat’ rule participants were required to press a button on the same side of the screen as the cat, for the ‘frog’ rule participants were required to press a button on the opposite side of the screen as the frog. Stage 1 contained 6 practice and 12 test trials, Stage 2 contained 4 practice and 12 test trials, and Stage 3 contained 33 test trials. We used the percentage of trials correctly completed for each stage as the outcome; Stages 1 and 2 rely on rule learning while Stage 3 relies on rule switching and inhibitory control. As piloting revealed some individuals showed response times that were too slow for the original version we amended the task to allow unlimited response times ( Startin *et al.*, unpublished observations). We also changed the cat colour to orange from white to contrast the green frog.

### Tests of motor coordination

***Finger-nose pointing.*** The finger-nose pointing test is a clinical measure of motor coordination ( Desrosiers *et al.*, 1995). Using the index finger on their dominant hand, participants alternatively pointed to the tip of their nose and a red circle with a 2cm diameter, 45cm away, as quickly as possible for 20 seconds. The outcome measure was the total number of times the participant pointed to the red circle.

***NEPSY-II – visuomotor precision.*** This task measures hand-eye coordination, and is taken from the Developmental NEuroPSYchological Assessment-II (NEPSY-II) ( Korkman *et al.*, 2007). Participants were timed as they traced train, car, and motorbike tracks (divided into squares), with a time limit of 180s for each track. The number of errors was calculated for each track (defined as those squares where the line went outside the track, there was a broken line due to a pen lift, or squares not completed in the time limit). Error scores and times were used to determine an overall score firstly for the train and car tracks combined and secondly the car and motorbike tracks combined using provided tables.

***ACTB – finger sequencing.*** The finger sequencing task is a measure of motor coordination. This task was adapted for the ACTB ( Edgin *et al.*, 2010) and administered to Cohort 2 only. Participants were required to tap a button as fast as possible using a variety of specified sequences, with a 10 second practice and 30 second test trial for each sequence. The total number of sequences completed was the main outcome used.

### Informant questionnaires

Informants completed a series of questionnaires about the participant while the participant was administered the cognitive battery. Informants were usually relatives or paid carers. Missing items from the DLD, OMQ and BRIEF-A were imputed for up to 15% of items within each domain by checking and imputing the nearest integer to the mean value of completed scores within that domain by hand. All reported measures for these questionnaires use the total scores including imputed values where relevant.

***Short ABS.*** The Short Adaptive Behavior Scale (short ABS) ( Hatton *et al.*, 2001), adapted from the Adaptive Behavior Scale – Residential and Community (Part I) ( Nihira *et al.*, 1993), recorded participants’ everyday adaptive abilities.

***DLD.*** The Dementia for Learning Disabilities (DLD) questionnaire is a measure of behaviours associated with cognitive decline in people with ID over the last two months ( Evenhuis, 1996).

***OMQ.*** The Observer Memory Questionnaire (OMQ) is an informant reported questionnaire relating to individuals’ memory abilities over the last two months ( O'Shea, 1996).

***BRIEF-A.*** The Behavior Rating Inventory of Executive Function – Adult version (BRIEF-A) ( Roth *et al.*, 2005) provides scores for informant reported problems with behaviours relating to executive functioning over the last month.

### Statistical analysis

The results presented here are limited to cross-sectional analyses of cognitive task data and related informant questionnaires. All statistical analyses were performed using SPSS version 22. We determined the number of individuals who completed each task, and for each outcome measure of interest calculated the mean, standard deviation and range of scores. As many variables deviated from normality as assessed using the Shapiro-Wilk test, with alpha set to p<0.01 to account for multiple comparisons, we also calculated medians and interquartile ranges. We determined the percentage of individuals at floor and ceiling level for each outcome of those who were able to complete the task (i.e. the number of individuals scoring the lowest and highest possible scores respectively). We compared responses between males and females in Cohort 2 using Student’s t-tests or Mann-Whitney U tests as appropriate. Correlation analyses were performed for Cohort 2 using Pearson’s correlation or Spearman’s rho as appropriate to assess concurrent validity and to determine the relationships between selected test scores; for these alpha was set to p<0.01 due to multiple comparisons. Absolute values of correlation coefficients of 0.70 and above were considered strong, between 0.50 and 0.69 were considered moderate, and between 0.30 and 0.49 were considered weak.

## Results

### Task completion and score distributions

Raw scores for KBIT-2 and total scores for informant questionnaires Cohort 1 KBIT-2 raw scores
Cohort 1 questionnaire total scores
Cohort 2 KBIT-2 raw scores
Cohort 2 questionnaire total scores Click here for additional data file. Copyright: © 2016 Startin CM et al. 2016 Data associated with the article are available under the terms of the Creative Commons Zero "No rights reserved" data waiver (CC0 1.0 Public domain dedication).

***Cohort 1: adults aged 36 years and over without dementia.*** Demographic information of 130 adults aged 36+ years without a clinical diagnosis of dementia is shown in Table 1. Nine (6.9%) participants were unable to undertake any tasks (one of whom did not understand English) and a further 12 (9.2%) participants did not pass the vision and hearing tests. All data relating to cognitive task completion and performance for this group are presented for 109 adults in Table 2, and data from informant questionnaires are shown in Table 3.

**Table 1.** Participant demographics across the groups.

	Adults aged 36+ without dementia	Adults aged 36+ with dementia	Adults aged 16–35
**Number**	130	51	124
**Age (mean±SD** **(range))**	47.77±7.01 (36–71)	54.20±6.95 (38–67)	25.24±5.53 (16–35)
**Sex**	74 males, 56 females	22 males, 29 females	59 males, 65 females
**ID severity** **(carer report)**	55 mild, 53 moderate, 22 severe	16 mild, 22 moderate, 8 severe, 5 unknown (NB pre-dementia)	48 mild, 63 moderate, 13 severe
**Ethnicity**	112 white, 4 Asian, 10 African, 3 mixed, 1 other	48 white, 2 Asian, 1 African	101 white, 6 Asian, 7 African, 7 mixed, 3 other

**Table 2.** Task completion rates and summary of results for main outcome measures for adults aged 36+ without dementia. ^a^ Significantly deviated from normality using Shapiro-Wilk test (P<0.010), ^b^ lower values indicate better performance, ^c^ 0 errors is at ceiling.

Test	Number completed	Reasons for non- completion	Outcome measure	Mean ± SD	Median (IQR)	Range	Number at floor	Number at ceiling
**KBIT-2**	105 (96.3%) verbal, 104 (95.4%) non- verbal	5 unable to complete	Verbal raw score	30.55 ± 17.47	28.00 (24.00)	2 – 80	0 (0.0%)	0 (0.0%)
			Performance raw score ^a^	12.55 ± 6.57	14.00 (7.00)	0 – 32	7 (6.7%)	0 (0.0%)
**CANTAB – PAL**	91 (83.5%)	10 refused 8 unable to complete	First trial memory score ^a^	7.00 ± 5.86	6.00 (11.00)	0 – 21	15 (16.5%)	0 (0.0%)
			Levels completed ^a^	4.98 ± 2.65	5.00 (6.00)	0 – 8	5 (5.5%)	23 (25.3%)
**CAMCOG – delayed** **incidental memory**	100 (91.7%)	7 refused 2 unable to complete	Object naming ^a^	5.65 ± 0.70	6.00 (1.00)	3 – 6	0 (0.0%)	75 (75.0%)
			Object recall ^a^	0.52 ± 1.05	0.00 (1.00)	0 – 6	72 (72.0%)	1 (1.0%)
			Object recognition ^a^	3.47 ± 1.85	4.00 (3.00)	0 – 6	6 (6.0%)	18 (18.0%)
**CAMCOG –** **orientation**	100 (91.7%)	6 refused 3 unable to complete	Total score ^a^	8.87 ± 3.56	10.50 (6.00)	0 – 12	1 (1.0%)	40 (40.0%)
**Delayed object** **memory**	97 (89.0%)	6 refused 4 unable to complete 2 technical problems	Immediate memory ^a^	9.09 ± 3.17	10.00 (5.00)	0 – 14	2 (2.1%)	4 (4.1%)
			Delayed memory ^a^	5.07 ± 1.80	5.00 (2.00)	0 – 7	3 (3.1%)	22 (22.7%)
**CANTAB – IED**	89 (81.7%)	14 refused 5 unable to complete 1 technical problems	Errors in stage 1 ^a^ ^b^	6.29 ± 9.17	2.00 (5.50)	0 – 33	0 (0.0%)	11 (12.4%) ^c^
			Levels completed ^a^	5.83 ± 3.07	7.00 (3.00)	0 – 9	13 (14.6%)	17 (19.1%)
**CANTAB – SRT**	84 (77.1%)	13 refused 7 unable to complete 5 technical problems	Total correct ^a^	87.07 ± 17.29	94.00 (17.25)	25 – 100	0 (0.0%)	13 (15.5%)
			Mean latency (ms) ^a^ ^b^	950.37 ± 480.55	853.78 (631.36)	311.33 – 2241.61	N/A	N/A
			Latency standard deviation (ms) ^b^	445.17 ± 216.29	426.63 (349.09)	45.32 – 980.98	N/A	N/A
**CAMCOG – verbal** **fluency**	101 (92.7%)	6 refused 2 unable to complete	Total animals named ^a^	8.40 ± 5.86	8.00 (8.00)	0 – 27	6 (5.9%)	N/A
**Tower of London**	97 (89.0%)	5 refused 6 unable to complete 1 technical problems	Total score ^a^	6.37 ± 3.23	8.00 (5.00)	0 – 10	8 (8.2%)	17 (17.5%)
**Finger-nose pointing**	98 (89.9%)	9 refused 2 unable to complete	Total completed ^a^	8.49 ± 4.83	8.00 (6.00)	0 – 23	2 (2.0%)	N/A
**NEPSY-II –** **visuomotor precision**	97 (89.0%) train and car, 96 (88.1%) car and motorbike	10 refused 2 unable to complete 1 technical problems	Train and car ^a^	13.52 ± 5.88	15.00 (8.00)	1 – 23	0 (0.0%)	0 (0.0%)
			Car and motorbike ^a^	11.45 ± 8.72	9.00 (15.00)	0 – 32	3 (3.1%)	0 (0.0%)

**Table 3.** Summary of results from informant questionnaires for adults aged 36+ without dementia. ^a^ Significantly deviated from normality using Shapiro-Wilk test (P<0.010), ^b^ higher scores indicate poorer abilities.

Questionnaire	Outcome measure	Number completed	Mean ± SD	Median (IQR)	Range	Number at floor	Number at ceiling
**Short ABS**	Total score	112 (86.2%)	71.89 ± 23.39	75.00 (38.50)	14 – 111	0 (0.0%)	0 (0.0%)
	Personal self- sufficiency ^a^	117 (90.0%)	26.74 ± 6.07	29.00 (6.00)	0 – 33	1 (0.9%)	14 (12.0%)
	Community self- sufficiency	115 (88.5%)	24.57 ± 12.06	24.00 (17.00)	0 – 47	1 (0.9%)	0 (0.0%)
	Personal-social responsibility ^a^	116 (89.2%)	20.78 ± 6.97	21.00 (10.75)	3 – 32	0 (0.0%)	1 (0.9%)
**DLD ^b^**	Sum of cognitive score ^a^	110 (84.6%)	12.23 ± 10.74	9.00 (16.50)	0 – 38	0 (0.0%)	12 (10.9%)
	Sum of social scores ^a^	113 (86.9%)	11.58 ± 7.75	11.00 (10.00)	0 – 36	0 (0.0%)	6 (5.3%)
**OMQ ^b^**	Total score	111 (85.4%)	82.50 ± 18.85	83.00 (26.00)	35 – 125	0 (0.0%)	0 (0.0%)
**BRIEF-A ^b^**	Total score	100 (76.9%)	122.11 ± 24.11	122.00 (37.50)	74 – 175	0 (0.0%)	0 (0.0%)
	Behavioural regulation index	117 (90.0%)	52.02 ± 11.21	51.00 (16.00)	30 – 80	0 (0.0%)	2 (1.7%)
	Metacognition index	101 (77.7%)	70.91 ± 14.71	72.00 (22.50)	43 – 100	0 (0.0%)	0 (0.0%)

Completion rates for each cognitive task in our battery were acceptable, approximately 90% for all non-computer tasks and 80% for computer tests. For those who completed the tasks many outcomes showed fewer than 10% of participants at floor and fewer than 20% of participants at ceiling. As anticipated, when converting KBIT-2 raw scores to IQ we found a high number of adults at floor, with 70 (66.7%) adults at floor for verbal IQ and 41 (39.4%) adults at floor for non-verbal IQ. The majority of outcomes from the informant questionnaires showed low floor and ceiling effects.

***Cohort 1: adults aged 36 years and over with dementia.*** Information about the demographics of 51 individuals with clinically diagnosed dementia is shown in Table 1. Of these, 22 had a diagnosis of AD, 1 a diagnosis of vascular dementia, 1 a diagnosis of dementia with Lewy bodies, and 27 had dementia of unspecified type. The mean age of dementia diagnosis was 51.70 years (SD 6.80, range 35–65 years), with a mean time since diagnosis of 2.46 years (SD 2.42, range 0–11 years). Of the adults in this group, 15 (29.4%) were unable to undertake any cognitive task with a further 9 (17.6%) failing the vision or hearing task. All data relating to cognitive task completion and performance for this group are presented for 27 individuals in Table 4, with data from informant questionnaires in Table 5.

**Table 4.** Task completion rates and summary of results for main outcome measures for adults aged 36+ with dementia. ^a^ Significantly deviated from normality using Shapiro-Wilk test (P<0.010), ^b^ lower values indicate better performance, ^c^ 0 errors is at ceiling.

Test	Number completed	Reasons for non- completion	Outcome measure	Mean ± SD	Median (IQR)	Range	Number at floor	Number at ceiling
**KBIT-2**	25 (92.6%) verbal, 24 (88.9%) non- verbal	3 unable to complete	Verbal raw score	18.68 ± 13.77	17.00 (24.00)	1 – 51	0 (0.0%)	0 (0.0%)
			Performance raw score	8.29 ± 6.45	8.00 (12.00)	0 – 19	4 (16.7%)	0 (0.0%)
**CANTAB – PAL**	20 (74.1%)	2 refused 5 unable to complete	First trial memory score ^a^	1.70 ± 2.58	0.50 (2.75)	0 – 9	10 (50.0%)	0 (0.0%)
			Levels completed	2.40 ± 2.09	2.00 (4.50)	0 – 6	5 (25.0%)	0 (0.0%)
**CAMCOG** **– delayed** **incidental** **memory**	25 (92.6%)	2 unable to complete	Object naming ^a^	5.40 ± 0.87	6.00 (2.00)	4 – 6	0 (0.0%)	16 (64.0%)
			Object recall ^a^	0.16 ± 0.47	0.00 (0.00)	0 – 2	22 (88.0%)	0 (0.0%)
			Object recognition	2.84 ± 1.70	2.00 (2.00)	0 – 6	1 (4.0%)	3 (12.0%)
**CAMCOG** **– orientation**	23 (85.2%)	3 unable to complete 1 technical problems	Total score	5.65 ± 3.92	5.00 (7.00)	0 – 12	2 (8.7%)	2 (8.7%)
**Delayed object** **memory**	21 (77.8%)	3 unable to complete 3 technical problems	Immediate memory ^a^	4.62 ± 4.12	3.00 (8.00)	0 – 11	5 (23.8%)	0 (0.0%)
			Delayed memory	2.67 ± 2.22	2.00 (5.00)	0 – 7	5 (23.8%)	1 (4.8%)
**CANTAB – IED**	20 (74.1%)	1 refused 6 unable to complete	Errors in stage 1 ^a b^	13.70 ± 12.91	7.00 (23.75)	0 – 39	0 (0.0%)	3 (15.0%) ^c^
			Levels completed ^a^	3.30 ± 3.39	1.50 (7.00)	0 – 8	8 (40.0%)	0 (0.0%)
**CANTAB – SRT**	17 (63.0%)	3 refused 7 unable to complete	Total correct	73.88 ± 18.83	72.00 (36.00)	41 – 99	0 (0.0%)	0 (0.0%)
			Mean latency (ms) ^b^	1293.65 ± 488.29	1160.93 (893.95)	588.00 – 2153.17	N/A	N/A
			Latency standard deviation (ms) ^b^	574.87 ± 166.43	609.07 (252.62)	219.57 – 815.67	N/A	N/A
**CAMCOG** ** – verbal fluency**	25 (92.6%)	2 unable to complete	Total animals named ^a^	5.00 ± 4.51	5.00 (7.00)	0 – 19	4 (16.0%)	N/A
**Tower of** **London**	16 (59.3%)	11 unable to complete	Total score	4.88 ± 3.79	5.50 (8.00)	0 – 10	3 (18.8%)	3 (18.8%)
**Finger-nose** **pointing**	23 (85.2%)	2 refused 2 unable to complete	Total completed ^a^	5.61 ± 5.09	3.00 (9.00)	0 – 15	2 (8.7%)	N/A
**NEPSY-II** **– visuomotor** **precision**	19 (70.4%) train and car, 18 (66.7%) car and motorbike	1 refused 8 unable to complete	Train and car	11.00 ± 7.57	11.00 (15.00)	0 – 21	1 (5.3%)	0 (0.0%)
			Car and motorbike	7.67 ± 7.90	5.00 (13.00)	0 – 24	4 (22.2%)	0 (0.0%)

**Table 5.** Summary of results from informant questionnaires for adults aged 36+ with dementia. ^a^ Significantly deviated from normality using Shapiro-Wilk test (P<0.010), ^b^ higher scores indicate poorer abilities.

Questionnaire	Outcome measure	Number completed	Mean ± SD	Median (IQR)	Range	Number at floor	Number at ceiling
**Short ABS**	Total score	43 (84.3%)	42.23 ± 24.51	38.00 (42.00)	3 – 92	0 (0.0%)	0 (0.0%)
	Personal self- sufficiency	43 (84.3%)	17.02 ± 9.70	17.00 (18.00)	0 – 33	1 (2.3%)	1 (2.3%)
	Community self- sufficiency ^a^	43 (84.3%)	11.98 ± 9.11	10.00 (15.00)	0 – 31	1 (2.3%)	0 (0.0%)
	Personal-social responsibility	43 (84.3%)	13.23 ± 7.32	13.00 (11.00)	1 – 28	0 (0.0%)	0 (0.0%)
**DLD ^b^**	Sum of cognitive score	42 (82.4%)	27.69 ± 10.53	29.00 (13.25)	3 – 44	1 (2.4%)	0 (0.0%)
	Sum of social scores	42 (82.4%)	23.93 ± 12.01	25.00 (22.00)	1 – 50	0 (0.0%)	0 (0.0%)
**OMQ ^b^**	Total score	37 (72.5%)	117.16 ± 13.82	119.00 (13.50)	78 – 142	0 (0.0%)	0 (0.0%)
**BRIEF-A ^b^**	Total score	33 (64.7%)	145.36 ± 31.54	149.00 (40.50)	77 – 199	0 (0.0%)	0 (0.0%)
	Behavioural regulation index	37 (72.5%)	57.22 ± 14.71	54.00 (21.00)	32 – 84	0 (0.0%)	0 (0.0%)
	Metacognition index	33 (64.7%)	88.24 ± 19.53	94.00 (30.00)	43 – 118	0 (0.0%)	0 (0.0%)

Completion rates for adults with dementia were lower than for those without dementia. Almost all tasks showed completion rates above 65%. For those able to complete the task the majority of outcomes showed fewer than 25% of individuals at floor and fewer than 15% of participants at ceiling. Again, we found high floor effects when converting KBIT-2 raw scores to IQ, with 21 (84.0%) adults at floor for verbal IQ and 15 (62.5%) adults at floor for non-verbal IQ. From the informant questionnaires, domains showed minimal floor and ceiling effects.

***Cohort 2: adults aged 16–35 years.*** Analyses were conducted for 124 adults aged 16–35 years. Demographic information is shown in Table 1. Of these, three (2.4%) did not pass the vision test, and so results relating to cognitive task performance for this group are presented for 121 individuals in Table 6 with data from informant questionnaires in Table 7.

**Table 6.** Task completion rates and summary of results for main outcome measures for adults aged 16–35. ^a^ Significantly deviated from normality using Shapiro-Wilk test (P<0.010), ^b^ lower values indicate better performance, ^c^ 0 errors is at ceiling.

Test	Number completed	Reasons for non- completion	Outcome measure	Mean ± SD	Median (IQR)	Range	Number at floor	Number at ceiling
**KBIT-2**	120 (99.2%) verbal, 121 (100.0%) non- verbal	1 unable to complete	Verbal raw score	35.03 ± 16.77	35.00 (23.00)	2 – 82	0 (0.0%)	0 (0.0%)
			Performance raw score ^a^	14.98 ± 6.90	16.00 (7.00)	0 – 32	5 (4.1%)	0 (0.0%)
**CANTAB – PAL**	108 (89.3%)	5 refused 7 unable to complete 1 technical problems	First trial memory score ^a^	10.22 ± 5.66	11.00 (7.75)	0 – 20	10 (9.3%)	0 (0.0%)
			Levels completed ^a^	6.29 ± 2.50	8.00 (2.00)	0 – 8	4 (3.7%)	56 (51.9%)
**CAMCOG** **– delayed** **incidental** **memory**	117 (96.7%)	1 refused 2 unable to complete 1 technical problems	Object naming ^a^	5.74 ± 0.68	6.00 (0.00)	2 – 6	0 (0.0%)	98 (83.8%)
			Object recall ^a^	1.19 ± 1.42	1.00 (2.00)	0 – 6	53 (45.3%)	1 (0.9%)
			Object recognition ^a^	4.30 ± 1.59	5.00 (3.00)	0 – 6	2 (1.7%)	34 (29.1%)
**CAMCOG** ** – orientation**	113 (93.4%)	1 refused 4 unable to complete 3 technical problems	Total score ^a^	9.65 ± 3.45	12.00 (4.00)	1 – 12	0 (0.0%)	65 (57.5%)
**Delayed object** **memory**	109 (90.1%)	2 refused 3 unable to complete 7 technical problems	Immediate memory ^a^	10.35 ± 2.83	11.00 (3.00)	0 – 14	2 (1.8%)	3 (2.8%)
			Delayed memory ^a^	5.84 ± 1.42	6.00 (2.00)	0 – 7	1 (0.9%)	43 (39.4%)
**Memory for** **sentences**	106 (87.6%)	2 refused 10 unable to complete 3 technical problems	Total words remembered ^a^	30.53 ± 13.68	33.50 (23.00)	3 – 49	0 (0.0%)	3 (2.8%)
**ACTB – virtual** **generated arena**	73 (60.3%)	4 refused 11 unable to complete 33 technical problems	Percentage of time spent in correct quadrant ^a^	26.52 ± 19.88	22.36 (21.04)	0.00 – 86.79	8 (11.0%)	0 (0.0%)
**CANTAB – IED**	109 (90.1%)	2 refused 6 unable to complete 4 technical problems	Errors in stage 1 ^a b^	4.19 ± 7.16	2.00 (3.00)	0 – 33	0 (0.0%)	19 (17.4%) ^c^
			Levels completed ^a^	6.57 ± 2.54	7.00 (1.00)	0 – 9	9 (8.3%)	22 (20.2%)
**CANTAB – SRT**	105 (86.8%)	3 refused 6 unable to complete 7 technical problems	Total correct ^a^	92.82 ± 13.30	98.00 (8.00)	13 – 100	0 (0.0%)	35 (33.3%)
			Mean latency (ms) ^a b^	692.48 ± 442.59	553.77 (462.81)	273.37 – 2500.61	N/A	N/A
			Latency standard deviation (ms) ^a b^	315.93 ± 208.43	274.23 (295.32)	32.94 – 950.49	N/A	N/A
**CAMCOG** **–verbal fluency**	114 (94.2%)	1 refused 5 unable to complete 1 technical problems	Total animals named	10.93 ± 5.84	10.50 (10.00)	0 – 24	3 (2.6%)	N/A
**Tower of London**	112 (92.6%)	3 unable to complete 6 technical problems	Total score ^a^	7.45 ± 2.90	8.00 (2.00)	0 – 10	6 (5.4%)	26 (23.2%)
**ACTB – cats and** **frogs**	86 (71.1%)	4 refused 3 unable to complete 28 technical problems	Stage 1 (cat rule alone) percentage correct ^a^	90.31 ± 18.57	100.00 (9.32)	33.33 – 100.00	0 (0.0%)	59 (68.6%)
			Stage 2 (frog rule alone) percentage correct ^a^	79.83 ± 27.12	95.83 (33.33)	0.00 – 100.00	2 (2.3%)	43 (50.0%)
			Stage 3 (combined rules) percentage correct ^a^	66.96 ± 21.64	56.16 (44.02)	33.33 – 100.00	0 (0.0%)	13 (15.1%)
**Finger-nose** **pointing**	116 (95.9%)	3 refused 2 technical problems	Total completed	11.01 ± 5.19	10.50 (8.00)	0 – 24	1 (0.9%)	N/A
**NEPSY-II** **– visuomotor** **precision**	118 (97.5%)	3 technical problems	Train and car ^a^	15.90 ± 5.26	18.00 (5.00)	2 – 23	0 (0.0%)	0 (0.0%)
			Car and motorbike ^a^	17.00 ± 9.61	18.00 (16.00)	0 – 40	1 (0.8%)	0 (0.0%)
**ACTB – finger** **sequencing**	83 (68.6%)	3 refused 10 unable to complete 25 technical problems	Total complete sequences	231.42 ± 62.36	241.00 (75.00)	30 – 369	0 (0.0%)	N/A

**Table 7.** Summary of results from informant questionnaires for adults aged 16–35. ^a^ Significantly deviated from normality using Shapiro-Wilk test (P<0.010), ^b^ higher scores indicate poorer abilities.

Questionnaire	Outcome measure	Number completed	Mean ± SD	Median (IQR)	Range	Number at floor	Number at ceiling
**Short ABS**	Total score ^a^	118 (95.2%)	79.03 ± 19.73	84.00 (28.50)	28 – 112	0 (0.0%)	0 (0.0%)
	Personal self- sufficiency ^a^	119 (96.0%)	28.91 ± 4.55	30.00 (6.00)	14 – 33	0 (0.0%)	31 (26.1%)
	Community self- sufficiency	119 (96.0%)	27.74 ± 10.36	29.00 (15.00)	4 – 47	0 (0.0%)	0 (0.0%)
	Personal-social responsibility ^a^	119 (96.0%)	22.53 ± 6.49	23.00 (10.00)	7 – 32	0 (0.0%)	5 (4.2%)
**DLD ^b^**	Sum of cognitive score ^a^	114 (91.9%)	7.57 ± 8.40	4.00 (11.00)	0 – 39	0 (0.0%)	23 (20.2%)
	Sum of social scores ^a^	118 (95.2%)	9.32 ± 6.85	8.50 (8.00)	0 – 31	0 (0.0%)	9 (7.6%)
**OMQ ^b^**	Total score	119 (96.0%)	74.82 ± 18.43	75.00 (23.00)	33 – 120	0 (0.0%)	0 (0.0%)
**BRIEF-A ^b^**	Total score	113 (91.1%)	121.03 ± 26.27	121.00 (31.00)	71 – 191	0 (0.0%)	0 (0.0%)
	Behavioural regulation index ^a^	117 (94.4%)	50.75 ± 12.32	49.00 (17.00)	31 – 82	0 (0.0%)	0 (0.0%)
	Metacognition index	113 (91.1%)	70.55 ± 16.71	70.00 (18.00)	40 – 116	0 (0.0%)	2 (1.8%)

We found high completion rates across the tasks in the battery, with the majority above 85% and many of the lower completion rates for some of the computer tasks being due to technical problems. For those who completed the tasks there were low floor effects, with many outcomes having fewer than 5% of participants at floor. Some outcomes however showed relatively high ceiling effects, though many were below 35%. When converting raw KBIT-2 scores to IQ we again found high floor effects, with 61 (50.8%) adults at floor for verbal IQ and 41 (33.9%) adults at floor for non-verbal IQ. The majority of domains from the informant questionnaires showed low floor and ceiling effects, although ceiling effects were found in over 20% of individuals for domains in the short ABS and DLD.

### Comparing scores for males and females in Cohort 2

There was no significant difference in age between males and females (t(122)=-0.854, p=0.395, males M 24.80 SD 5.79, females M 25.65 SD 5.29, 95% CI (-2.82, 1.12)). Females showed significantly better performance on the verbal subtests of the KBIT-2 (t(109.5)=-2.15, p=0.034, 95% CI (-12.40, -0.50)). For the informant questionnaires females showed better cognitive abilities as assessed by the DLD cognitive domain (p=0.041). There were no other significant differences in performance between males and females (all p>0.05; see Table 8 and Table 9). Within Cohort 2 there were no significant correlations with age for any cognitive test outcomes or informant questionnaire scores (all p>0.05; see Table 10 and Table 11).

**Table 8.** Comparing cognitive test scores between males and females for adults aged 16–35. All group comparisons used Mann Whitney U tests aside from ^a^ when Student’s t-tests were used as data did not deviate from normality.

	Mean ± SD		Median (IQR)		
	Males	Females	Males	Females	p
**KBIT-2 verbal score** ^a^	31.75 ± 13.68	38.20 ± 18.87	32.00 (16.00)	38.00 (28.00)	0.034
**KBIT-2 non-verbal score**	14.97 ± 6.90	15.00 ± 6.95	16.00 (7.00)	16.00 (7.00)	0.845
**PAL first trial memory score**	10.04 ± 5.17	10.39 ± 6.12	10.00 (7.00)	12.50 (11.00)	0.444
**PAL levels completed**	6.35 ± 2.37	6.23 ± 2.63	7.50 (2.00)	8.00 (3.50)	0.806
**CAMCOG object naming**	5.75 ± 0.61	5.74 ± 0.75	6.00 (0.00)	6.00 (0.00)	0.702
**CAMCOG object recall**	1.11 ± 1.34	1.26 ± 1.49	1.00 (2.00)	1.00 (2.00)	0.657
**CAMCOG object recognition**	4.07 ± 1.72	4.51 ± 1.45	4.50 (4.00)	5.00 (2.00)	0.207
**CAMCOG orientation**	9.18 ± 3.59	10.10 ± 3.29	11.00 (6.00)	12.00 (3.00)	0.086
**Object memory immediate**	10.30 ± 2.48	10.39 ± 3.15	11.00 (3.00)	11.00 (2.00)	0.367
**Object memory delayed**	5.91 ± 1.15	5.79 ± 1.65	6.00 (2.00)	6.00 (2.00)	0.664
**Memory for sentences**	29.16 ± 13.33	31.80 ± 14.00	33.00 (22.00)	34.00 (24.00)	0.267
**IED errors stage 1**	3.62 ± 6.12	4.72 ± 8.01	1.50 (3.00)	2.00 (3.00)	0.874
**IED levels complete**	6.88 ± 2.28	6.28 ± 2.74	7.00 (1.00)	7.00 (0.50)	0.212
**SRT total correct**	92.53 ± 15.24	93.12 ± 11.13	98.00 (8.50)	98.00 (7.50)	0.478
**SRT mean latency (ms)**	708.70 ± 517.62	675.95 ± 354.58	491.76 (485.52)	590.04 (442.00)	0.497
**SRT latency standard deviation (ms)**	293.94 ± 199.77	338.34 ± 216.53	244.95 (300.57)	319.94 (300.64)	0.290
**CAMCOG verbal fluency** ^a^	10.52 ± 6.02	11.33 ± 5.68	10.00 (10.00)	12.00 (8.00)	0.462
**Tower of London**	7.64 ± 2.70	7.27 ± 3.08	8.00 (2.00)	9.00 (3.00)	0.879
**Cats and frogs Stage 1**	87.60 ± 21.42	92.89 ± 15.18	100.00 (18.18)	100.00 (8.33)	0.284
**Cats and frogs Stage 2**	80.36 ± 27.45	79.33 ± 27.11	91.67 (33.33)	100.00 (41.25)	0.952
**Cats and frogs Stage 3**	65.49 ± 20.03	68.37 ± 23.22	54.86 (35.79)	59.43 (48.00)	0.497
**Finger nose pointing** ^a^	10.88 ± 5.48	11.14 ± 4.94	11.00 (8.00)	10.00 (8.00)	0.790
**NEPSY-II visuomotor precision** **train and car**	15.83 ± 4.97	15.97 ± 5.57	18.00 (5.00)	18.00 (5.00)	0.528
**NEPSY-II visuomotor precision car** **and motorbike** ^a^	17.38 ± 9.83	16.63 ± 9.46	18.00 (17.00)	18.00 (15.00)	0.675
**Finger sequencing** ^a^	238.08 ± 65.57	225.23 ± 59.31	250.50 (80.25)	241.00 (68.00)	0.352

**Table 9.** Comparing informant scores between males and females for adults aged 16–35. All group comparisons used Mann Whitney U tests aside from ^a^ when Student’s t-tests were used as data did not deviate from normality.

	Mean ± SD		Median (IQR)		
	Males	Females	Males	Females	p
**Short ABS Total score**	77.69 ± 20.34	80.17 ± 19.29	79.50 (35.25)	84.50 (22.00)	0.563
**Short ABS Personal self-** **sufficiency**	28.95 ± 4.66	28.88 ± 4.48	31.00 (7.00)	30.00 (4.00)	0.704
**Short ABS Community** **self-sufficiency** ^a^	27.24 ± 10.24	28.17 ± 10.53	27.00 (17.00)	30.00 (14.50)	0.625
**Short ABS Personal-** **social responsibility**	21.84 ± 7.01	23.13 ± 5.99	22.00 (12.00)	25.00 (7.00)	0.384
**DLD Sum of cognitive** **score**	9.00 ± 8.74	6.28 ± 7.95	5.00 (14.00)	3.00 (8.75)	0.041
**DLD Sum of social** **scores**	9.16 ± 6.50	9.47 ± 7.20	8.50 (8.00)	8.50 (6.50)	0.985
**OMQ Total score** ^a^	77.93 ± 19.24	72.16 ± 17.42	79.00 (26.00)	71.50 (20.25)	0.089
**BRIEF-A Total score** ^a^	121.29 ± 28.01	120.81 ± 24.98	118.00 (41.00)	121.50 (24.25)	0.922
**BRIEF-A Behavioural** **regulation index** ^a^	50.83 ± 12.59	50.68 ± 12.19	49.00 (18.25)	49.00 (16.00)	0.948
**BRIEF-A Metacognition** **index** ^a^	71.33 ± 18.14	69.90 ± 15.56	71.00 (25.00)	70.00 (15.50)	0.653

**Table 10.** Correlations between cognitive test outcome scores and age across adults aged 16–35. Values given are correlation coefficients (p values); *p<0.05, **p<0.01, ***p<0.001. All correlations used were Spearman’s rho apart from ^a^ when Pearson’s correlation was used as data do not deviate from normality. Values in italics represent correlation coefficients greater than 0.50.

	KBIT-2 verbal score	KBIT-2 non- verbal score	PAL first trial memory score	Object memory immediate	Object memory delayed	Memory for sentences	Arena	IED levels complete	SRT mean latency	SRT latency standard deviation
**KBIT-2** **verbal score**	-	0.651*** (<0.001)	0.581*** (<0.001)	0.488*** (<0.001)	0.394*** (<0.001)	0.827*** *(<0.001)*	0.133 (0.262)	0.409*** (<0.001)	-0.506*** (<0.001)	-0.583*** (<0.001)
**KBIT-2** **non-verbal** **score**	-	-	0.636*** (<0.001)	0.448*** (<0.001)	0.460*** (<0.001)	0.510*** *(<0.001)*	0.243* (0.039)	0.387*** (<0.001)	-0.530*** (<0.001)	-0.533*** (<0.001)
**PAL** **first trial** **memory** **score**	-	-	-	0.522*** (<0.001)	0.450*** (<0.001)	0.467*** (<0.001)	0.171 (0.157)	0.392*** (<0.001)	-0.489*** (<0.001)	-0.614*** (<0.001)
**Object** **memory** **immediate**	-	-	-	-	0.536*** (<0.001)	0.342*** (<0.001)	0.043 (0.728)	0.220* (0.027)	-0.358*** (<0.001)	-0.405*** (<0.001)
**Object** **memory** **delayed**	-	-	-	-	-	0.264** (0.008)	0.115 (0.353)	0.174 (0.083)	-0.238* (0.020)	-0.250* (0.014)
**Memory for** **sentences**	-	-	-	-	-	-	0.172 (0.155)	0.256* (0.011)	-0.387*** (<0.001)	-0.391*** (<0.001)
**Arena**	-	-	-	-	-	-	-	0.108 (0.371)	-0.265* (0.028)	-0.223 (0.065)
**IED levels** **complete**	-	-	-	-	-	-	-	-	-0.288** (0.004)	-0.366*** (<0.001)
**SRT mean** **latency**	-	-	-	-	-	-	-	-	-	0.888*** (<0.001)
**SRT latency** **standard** **deviation**	-	-	-	-	-	-	-	-	-	-
	**Verbal fluency**	**Tower of London**	**Cats and frogs Stage 3**	**Finger nose pointing**	**NEPSY-II visuomotor precision train and car**	**NEPSY-II visuomotor precision car and motorbike**	**Finger sequencing**	**Age**
**KBIT-2** **verbal score**	0.694*** (<0.001) ^a^	0.429*** (<0.001)	0.568*** (<0.001)	0.592*** (<0.001) ^a^	0.407*** (<0.001)	0.515*** (<0.001)	0.375*** (<0.001) ^a^	0.040 (0.662)
**KBIT-2** **non-verbal** **score**	0.503*** (<0.001)	0.380*** (<0.001)	0.541*** (<0.001)	0.563*** (<0.001)	0.323*** (<0.001)	0.502*** (<0.001)	0.490*** (<0.001)	-0.107 (0.240)
**PAL** **first trial** **memory** **score**	0.442*** (<0.001)	0.522*** (<0.001)	0.495*** (<0.001)	0.584*** (<0.001)	0.468*** (<0.001)	0.539*** (<0.001)	0.334** (0.003)	-0.181 (0.060)
**Object** **memory** **immediate**	0.430*** (<0.001)	0.261** (0.008)	0.277* (0.012)	0.385*** (<0.001)	0.207* (0.032)	0.380*** (<0.001)	0.250* (0.027)	0.038 (0.692)
**Object** **memory** **delayed**	0.281** (0.003)	0.298** (0.002)	0.302** (0.006)	0.291** (0.002)	0.341*** (<0.001)	0.428*** (<0.001)	0.121 (0.293)	0.002 (0.980)
**Memory for** **sentences**	0.593*** (<0.001)	0.229* (0.022)	0.482*** (<0.001)	0.373*** (<0.001)	0.226* (0.020)	0.334*** (<0.001)	0.334** (0.002)	-0.089 (0.360)
**Arena**	0.149 (0.214)	-0.047 (0.699)	0.066 (0.598)	0.126 (0.288)	-0.010 (0.934)	0.075 (0.526)	0.284* (0.017)	-0.005 (0.968)
**IED levels** **complete**	0.372*** (<0.001)	0.302** (0.002)	0.305** (0.006)	0.406*** (<0.001)	0.241* (0.012)	0.286** (0.003)	0.194 (0.085)	-0.048 (0.621)
**SRT mean** **latency**	-0.426*** (<0.001)	-0.271** (0.006)	-0.406*** (<0.001)	-0.539*** (<0.001)	-0.296** (0.002)	-0.402*** (<0.001)	-0.628*** (<0.001)	0.115 (0.239)
**SRT latency** **standard** **deviation**	-0.472*** (<0.001)	-0.433*** (<0.001)	-0.485*** (<0.001)	-0.572*** (<0.001)	-0.320** (0.001)	-0.438*** (<0.001)	-0.608*** (<0.001)	0.036 (0.714)
**Verbal** **fluency**	-	0.331*** (<0.001)	0.391*** (<0.001)	0.600*** (<0.001) ^a^	0.461*** (<0.001)	0.476*** (<0.001)	0.428*** (<0.001) a	-0.059 (0.529)
**Tower of** **London**	-	-	0.235* (0.034)	0.389*** (<0.001)	0.410*** (<0.001)	0.505*** (<0.001)	0.343** (0.002)	-0.024 (0.797)
**Cats and** **frogs Stage 3**	-	-	-	0.379*** (<0.001)	0.311** (0.004)	0.360** (0.001)	0.252* (0.030)	0.029 (0.792)
**Finger nose** **pointing**	-	-	-	-	0.530*** (<0.001)	0.524*** (<0.001)	0.586*** (<0.001) a	-0.083 (0.373)
**NEPSY-II** **visuomotor** **precision** **train and car**	-	-	-	-	-	0.701*** (<0.001)	0.383*** (<0.001)	-0.043 (0.637)
**NEPSY-II** **visuomotor** **precision** **car and** **motorbike**	-	-	-	-	-	-	0.376*** (<0.001)	-0.014 (0.877)
**Finger** **sequencing**	-	-	-	-	-	-	-	-0.061 (0.583)

**Table 11.** Correlations between cognitive test outcome scores and age across adults aged 16–35. Values given are correlation coefficients (p values); *p<0.05, **p<0.01, ***p<0.001. All correlations used were Spearman’s rho apart from ^a^ when Pearson’s correlation was used as data do not deviate from normality. Values in italics represent correlation coefficients greater than 0.50.

	Short ABS Personal self-sufficiency	Short ABS Community self- sufficiency	Short ABS Personal-social responsibility	OMQ Total score	BRIEF-A Behavioural regulation index	BRIEF-A Metacognition index	Age
**Short ABS** **Personal self-** **sufficiency**	-	0.719*** (<0.001)	0.687*** (<0.001)	-0.426*** (<0.001)	-0.380*** (<0.001)	-0.549*** (<0.001)	0.111 (0.230)
**Short ABS** **Community self-** **sufficiency**	-	-	0.762*** (<0.001)	-0.535*** (<0.001) ^a^	-0.457*** (<0.001)	-0.669*** (<0.001) ^a^	0.162 (0.077)
**Short ABS** **Personal-social** **responsibility**	-	-	-	-0.631*** (<0.001)	-0.572*** (<0.001)	-0.731*** (<0.001)	0.087 (0.346)
**OMQ Total score**	-	-	-	-	0.488*** (<0.001)	0.741*** (<0.001) ^a^	-0.122 (0.187)
**BRIEF-A** **Behavioural** **regulation index**	-	-	-	-	-	0.643*** (<0.001)	0.070 (0.452)
**BRIEF-A** **Metacognition** **index**	-	-	-	-	-	-	-0.125 (0.187)

### Correlations between outcome scores for Cohort 2

The majority of cognitive test outcomes showed significant correlations with all other outcomes in the battery (p<0.01), with the exception of the computer generated arena which showed no significant correlations at the p<0.01 level ( Table 10). All outcomes from the informant questionnaires showed significant correlations with each other (see Table 11). Due to a high number of adults aged 16–35 scoring at or close to ceiling in the DLD domains these scores were not included in correlational analyses. To better investigate the relationships between test outcomes we considered the absolute values of correlation coefficients.

Moderate and strong correlations revealed four clusters of test outcomes within our cognitive data. One cluster contained PAL first trial memory score and object memory immediate score (r=0.522), suggesting this is a visuospatial memory cluster. Another contained SRT mean latency and latency standard deviation, finger-nose pointing and finger sequencing (0.539<r<0.628), suggesting this is a motor coordination cluster. The next contained memory for sentences and verbal fluency (r=0.593). These two tasks also correlated highly with KBIT-2 verbal and non-verbal scores (0.503<r<0.827), in particular the former, suggesting this represents a language cluster. The final cluster contained outcomes that were not all necessarily related to each other but were related to at least two other outcomes in the cluster; this consisted of PAL first trial memory score, SRT mean latency and latency standard deviation, Tower of London, finger-nose pointing and NEPSY-II visuomotor precision car and motorbike (0.271<r<0.614). Again, most of this cluster correlated with KBIT-2 verbal and non-verbal scores (0.380<r<0.636). This cluster may be related to processing speed and sustained attention. Finally, the cats and frogs Stage 3 score also correlated highly with KBIT-2 verbal and non-verbal scores (r=0.568 and r=0.541 respectively), suggesting performance on this task is highly related to general abilities.

Within the informant questionnaire outcomes the best correlations were between subscales related to complex adaptive functioning such as personal-social responsibility and higher cognitive functions (Short ABS Personal-social responsibility and OMQ r=-0.631, Short ABS Personal-social responsibility and BRIEF-A Metacognition index r=-0.731).

## Discussion

Here we describe a cognitive test battery to provide detailed assessment of cognitive abilities in individuals with DS, along with data for test completion and outcomes. We deliberately assessed individuals with a wide range of ages and ID severities and those with and without a clinical diagnosis of dementia, in order to provide cognitive test data that is representative of the adult population with DS. Results from individuals without dementia suggest high completion rates across the tasks. Computer-based tasks had lower completion rates, in some cases (up to 27.3%) due to technical issues. Completion rates for those with dementia were lower, with approximately half of individuals unable to undertake any task. Our outcome measures for each task and informant measure showed a range of scores, with many showing low floor and ceiling effects. Non-verbal age-adjusted IQ scores had lower floor effects than verbal IQ scores for all groups.

Females aged 16–35 years performed better than males on general verbal abilities, and also showed better cognitive abilities as assessed by the DLD cognitive domain. We identified clusters of cognitive test scores within our battery relating to visuospatial memory, motor coordination, language abilities, and processing speed / sustained attention.

Our results show a wide range of individuals’ cognitive abilities, and suggest our battery is suitable for a wide range of adults with DS. Our future studies will use our baseline results presented here to investigate cognitive abilities and changes in cognitive abilities associated with ageing and dementia. Individual differences in the dementia phenotype and cognitive profiles of people with DS emphasises the importance of studying factors contributing towards these variations ( Karmiloff-Smith *et al.*, 2016). We will also investigate factors including genetic, medical and socioeconomic variations that may be associated with these abilities. We hope our results will help identify risk and protective factors for the development of dementia in people with DS, and factors relating to baseline cognitive abilities. This will aid identifying relevant potential mechanisms and predictive phenotypes, and may help to inform interventions that can influence developmental trajectories.

### Final test and outcome selection

Many of the tests within our battery show a range of scores with low floor and ceiling effects and high validity, as determined by exploring relationships between outcomes in Cohort 2. However, several tests within our battery may have limited use based on our study aims. Firstly, the CAMCOG incidental memory test may not be useful, with high floor effects for the recall score for all groups. Future longitudinal studies will determine if this is a useful test to assess cognitive decline within individuals. Secondly, a previous pilot study suggested the virtual generated arena is not useful in older adults ( Sinai & Strydom, unpublished observations), and our current analyses showed that for younger adults the test scores showed limited correlations with other task measures. Further, both the mean and median times spent in the correct quadrant were approximately 25%, and as individuals should spend 25% of their time in the correct quadrant by chance alone this suggests this measure is not useful.

As expected, when converting raw scores on the KBIT-2 to age-dependent IQ scores we found high floor effects across all participant groups. IQ score floor effects were lower for non-verbal IQ than verbal IQ in all our groups. Age-dependent non-verbal IQ scores may therefore be more useful than verbal IQ scores for future studies, and also offer an advantage if comparing individuals or studies across language groups.

The ideal test and outcome measure to use in neuropsychological research depends upon the cognitive ability of interest, the specific research question and population assessed, in addition to floor/ceiling effects and the spread of results observed. Within different age cohorts and for our different research questions different tests and outcome measures will therefore be useful, in particular as score ranges and floor and ceiling effects varied across groups (e.g. to assess cognitive decline then outcomes with low floor effects prior to the onset of decline are essential). For several cognitive tasks within our battery, in particular the CANTAB tasks, there are multiple outcome measures, and we have identified those outcomes that will be most useful in our future studies (see Box 1).

Box 1. **Ideal outcome measures to use in future studies**

**Table:** 

Test	Outcome measure	Comments
PAL	First trial memory score	Ideal for younger adults as wide range with no ceiling effect
	Number of stages complete	Ideal for older adults as small floor effect
IED	Number of stages completed	Ideal for younger adults as can identify subgroups who can complete extra-dimensional shift and who cannot pass any levels
SRT	Latency standard deviation	Ideal for older and younger adults to measure attention as no floor or ceiling effect and accounts for individual variations in motor coordination
Object memory	Immediate memory score	Ideal for younger adults as small ceiling effect and wide range of scores
NEPSY-II visuomotor precision	Car and motorbike score	Ideal for older and younger adults as wide range
Cats and frogs	Stage 3	Ideal for younger adults as small ceiling effect and can identify subgroup able to follow both rules

A high proportion of individuals with dementia were unable to complete any cognitive tests. For those able to undertake cognitive tasks completion rates were generally higher for table-top tasks compared to computer tasks. This suggests the use of some longer computer tasks may not be suitable for an older population at risk for dementia, and instead may need to be replaced with traditional table-top tasks and informant questionnaires. We also noted that in many adults with dementia and in some adults aged 36+ years without dementia attention levels appeared to negatively affect task performance. A similar observation was made by Sinai *et al.* (2016), and future test batteries should account for this.

Finally, during data collection we found some questions within two of the informant questionnaires used, the BRIEF-A and OMQ, were often unsuitable for older adults and those with more severe IDs. As a result, we developed a new informant questionnaire, the Cognitive Scale for Down Syndrome (CS-DS), to assess cognitive abilities in people with DS, focusing on executive function, memory and language abilities. This questionnaire showed high reliability and validity ( Startin *et al.*, 2016a).

### Validity of the test battery

The majority of cognitive test scores correlated well with all other cognitive test scores in adults aged 16–35. It has previously been proposed that cognitive measures are more highly correlated in those with lower compared to higher IQs ( Detterman & Daniel, 1989). The high correlations between test scores and KBIT-2 raw scores indicate that higher general abilities are related to better individual task performance, and it has similarly been suggested the high variability in neurocognitive task performance in people with DS is due to variability in IQ ( de Sola *et al.*, 2015). Further, de Sola *et al.* (2015) and Liogier d'Ardhuy *et al.* (2015) found better task performance in individuals with higher IQs.

To determine clusters of related cognitive outcomes in adults aged 16–35 before the onset of cognitive decline we examined correlational coefficients of 0.50 and above. We identified the presence of clusters relating to visuospatial memory, motor coordination, language abilities, and sustained attention/processing speed. These results suggest the presence of related cognitive abilities in this population that could inform further development of outcome measures.

### Effect of sex on task performance

We found females scored higher for KBIT-2 verbal scores and for informant report for the DLD cognitive domain than males in adults aged 16–35 years. Previous studies have also reported higher linguistic abilities in females compared to males ( de Sola *et al.*, 2015; Liogier d'Ardhuy *et al.*, 2015), in addition to better performance on tasks of memory, executive function and attention (including the PAL and SRT) ( de Sola *et al.*, 2015) and higher functional abilities ( Lund, 1988; Määttä *et al.*, 2006). The effect of gender on cognitive and functional abilities in DS requires further study.

### Possible effect of cognitive decline and ageing on task performance

We found no significant correlations with age and cognitive test outcomes or informant questionnaire scores in adults aged 16–35. Performance was however generally poorer in adults aged 36+ compared to those aged 16–35. Our future analyses will focus on the impact of cognitive decline and ageing on abilities in individuals with DS.

Previous studies have confirmed poorer performance on many of the cognitive tasks within our battery for adults with cognitive decline or dementia compared to those with no decline ( Adams & Oliver, 2010; Ball *et al.*, 2008; Oliver *et al.*, 2005; Sinai *et al.*, 2016). Previous studies have also found poorer performance associated with ageing in DS for the PAL ( Crayton *et al.*, 1998; Oliver *et al.*, 2005) and Tower of London ( Ghezzo *et al.*, 2014). These results suggest our battery should be sensitive to the presence of dementia and many of our tasks may be useful for predicting and tracking cognitive decline. Cognitive abilities and changes in these individuals over the course of our longitudinal study will be of particular interest when determining the effects of age-related and dementia-related changes in cognition.

### Strengths and limitations

A major strength of our study and analyses is the large sample size, including a wide variety of ages and ID severities, and both those with and without a clinical diagnosis of dementia. We recruited individuals from a variety of settings, including volunteers and local ID clinical teams, suggesting our sample should be representative of individuals with DS in the UK.

Our results suggest the majority of our tasks have high completion rates for adults who do not have a diagnosis of dementia, with test scores showing a wide range and select outcomes showing low floor and ceiling effects. The battery will therefore be largely suitable for further analyses to assess cognitive decline, dementia, ageing, and baseline cognitive abilities in adults with DS.

For adults with a diagnosis of dementia completion rates were much lower however, although this population will always be difficult to assess with psychometric tests. For adults unable to complete any of the tasks in the battery informant ratings of abilities are invaluable, although further work is needed to determine the relationships between cognitive test scores and informant measure outcomes. A further limitation lies with the use of KBIT-2 IQ scores, which showed a high number of individuals at floor level, similar to other IQ tests in this population. For this reason we chose to use raw scores as the main outcome for the KBIT-2.

## Conclusion

We report a cognitive battery and related informant measures to assess general abilities, memory, executive function, and motor coordination abilities in individuals with DS. We assessed participants with a range of ages and abilities, and our results suggest the battery is suitable for the majority of adults with DS. Many test outcomes showed a range of scores with low floor and ceiling effects. This battery will be used in our future studies to assess factors influencing individual differences in cognitive decline, dementia, ageing, and baseline cognitive abilities in adults with DS.

## Data availability

The data referenced by this article are under copyright with the following copyright statement: Copyright: © 2016 Startin CM et al.

Data associated with the article are available under the terms of the Creative Commons Zero "No rights reserved" data waiver (CC0 1.0 Public domain dedication). 

*Figshare:* Raw scores for KBIT-2 and total scores for informant questionnaires doi: 10.6084/m9.figshare.4206429.v1 ( Startin *et al.*, 2016b).

## Supplementary material

**Supplementary File 1**. Summary table of assessments for general abilities, memory, executive function, and motor coordination, with outcomes of interest.

**Supplementary File 2**. Assessment schedule for cognitive test battery.
