# Supplementary material for: The LonDownS adult cognitive assessment to study cognitive abilities and decline in Down syndrome
Source: Wellcome Open Res. 2016 Nov 15;1:11. [Version 1] doi: 10.12688/wellcomeopenres.9961.1 (PMC5176078; doi:10.12688/wellcomeopenres.9961.1)
Supplement: Supplementary file 2 [file wellcomeopenres-1-10736-s0001.tgz › 292b132f-0c4d-42c2-9d32-7c2ed36f9415.pdf]

Test name	Test type	Description	Primary ability assessed	Outcomes and score ranges
Kaufman Brief Intelligence Test 2 (KBIT-2)	Table top test	Participants are required to identify correct answers in two verbal subtests (verbal knowledge and riddles) plus one non-verbal subtest (matrices).	General cognitive abilities	Verbal raw score (0-108); Non-verbal raw score (0-46)
Short Adaptive Behavior Scale (Short ABS)	Informant questionnaire	Questions relate to adaptive behaviours. Contains 3 domains.	Adaptive behaviours	Total score (0-113); Personal self-sufficiency (0-33); Community self-sufficiency (0-48); Personal-social responsibility (0-32)
Dementia for Learning Disabilities (DLD)	Informant questionnaire	Questions relate to behaviours associated with cognitive decline over the past 2 months. Contains 2 domains.	Cognitive decline	Cognitive abilities (0-44); Social abilities (0-60)

Assessments of general abilities

Test name	Test type	Description	Primary ability assessed	Outcomes
CANTAB – paired associates learning (PAL)	Computer test	Participants observe and recall pattern locations.	Visuospatial short term memory	First trial memory score (0-26); Number of stages completed (0-8)
CAMCOG – delayed incidental memory	Table top test	Participants name pictures of objects. Following two distractor tasks participants freely recall the objects then identify them based on recognition.	Short term memory	Object naming (0-6); Object recall (0-6); Object recognition (0-6)
CAMCOG – orientation	Table top test	Participants are asked their full name, the day of the week, the month, the year, where they are, and the nearest city / town. Clues given (for a lower score) if incorrect / no response.	Orientation (knowledge of when it is and where they are)	Total score (0-12)

Delayed object memory	Table top test	For each trial objects are named by participants, then recalled in two immediate trials plus one 5 minute delayed trial.	Short term memory	Immediate recall (0-14); Delayed recall (0-7)
NAID – memory for sentences	Table top test	Participants repeat sentences after researcher (6 sentences with two attempts each).	Verbal short term memory	Words remembered (0-49)
ACTB – virtual generated arena	Computer test	Participants learn then locate where a hidden carpet is in a virtual room, using visual cues.	Visuospatial short term memory	Percentage of time of final trial searching in correct quadrant (0-100%)
Observer Memory Questionnaire (OMQ)	Informant questionnaire	Questions relate to individuals' memory abilities over the past 2 months.	Memory abilities	Total score (30-150)

Assessments of memory

Test name	Test type	Description	Primary ability assessed	Outcomes
CANTAB – intra/extra dimensional set shift (IED)	Computer test	Participants are presented with two patterns and required to select the 'correct' one according to a rule. When a rule has been established there is a rule change.	Rule learning and set shifting	Number of stages completed (0-9); Number of stage 1 errors (0-50)
CANTAB – simple reaction time (SRT)	Computer test	Reaction time test requiring participants to press a button in response to a white square appearing.	Attention / motor abilities	Response time standard deviation (N/A); Total number of correct responses (0-100); Mean response time (N/A)
Verbal fluency	Table top test	Participants are asked to name as many animals as they can in 1 minute.	Verbal fluency	Number of unique animals (0-N/A)
Tower of London	Table top test	Participants move beads on a board to match presented configurations.	Working memory and planning	Total score (0-10)

ACTB – cats and frogs	Computer test	Participants are required to learn two rules about which button to press when they see a cat or frog on the screen.	Rule learning and switching, inhibitory control, and working memory	Percentage of correct trials Stage 1 (cat rule) (0-100%); Percentage of correct trials Stage 2 (frog rule) (0-100%); Percentage of correct trials Stage 3 (cat and frog rules combined) (0-100%)
Behavior Rating Inventory of Executive Function – Adult version (BRIEF-A)	Informant questionnaire	Questions relate to problems with behaviours relating to executive functioning over the past month. Contains 2 domains.	Executive function	Total score (70-210); Behavioural regulation index (30-90); Metacognition index (40-120)

Assessments of executive function

Test name	Test type	Description	Primary ability assessed	Outcomes
Finger-nose pointing	Table top test	Participants alternately touch the tip of their nose and a red circle 45cm away for 20s.	Motor coordination	Total number of times the circle is touched (0-N/A)
NEPSY-II – visuomotor precision	Table top test	Participants draw around three tracks (train, car, motorbike). Times (max 180s) and errors for each track are used to calculate overall scores.	Motor coordination	Train and car score (0-30); Car and motorbike score (0-52)
ACTB – finger sequencing	Computer test	Participants are required to tap a button as fast as possible in specified finger sequences.	Motor coordination	Total number of sequences completed (0-N/A)

Assessments of motor coordination
