# Supplementary material for: The LonDownS adult cognitive assessment to study cognitive abilities and decline in Down syndrome
Source: Wellcome Open Res. 2016 Nov 15;1:11. [Version 1] doi: 10.12688/wellcomeopenres.9961.1 (PMC5176078; doi:10.12688/wellcomeopenres.9961.1)
Supplement: Supplementary file 3 [file wellcomeopenres-1-10736-s0002.tgz › bfa127f4-cb8c-4c81-8967-95238558c7aa.pdf]

Assessment schedule

The order of the assessments performed is shown in Box 1. We used a counter-balanced approach, alternating by participant recruitment number.

**Assessment schedule for adults aged 36+ years odd numbers**	**Assessment schedule for adults aged 36+ years even numbers**
Kay vision test<br>Whisper hearing test<br>KBIT-2<br>CANTAB - MOT<br>CANTAB - PAL<br>Finger nose pointing<br>CAMCOG - Delayed incidental memory, verbal fluency and orientation<br>Break<br>CANTAB - IED<br>Delayed object memory with gait and measurements in the break for delayed recall<br>CANTAB - SRT<br>Tower of London<br>NEPSY-II - visuomotor precision	Kay vision test<br>Whisper hearing test<br>KBIT-2<br>NEPSY-II - visuomotor precision<br>Tower of London<br>CANTAB - SRT<br>Delayed object memory with gait and measurements in the break for delayed recall<br>Break<br>CANTAB - MOT<br>CANTAB - IED<br>CAMCOG - Delayed incidental memory, verbal fluency and orientation<br>Finger nose pointing<br>CANTAB - PAL
**Assessment schedule for adults aged 16-35 years odd numbers**	**Assessment schedule for adults aged 16-35 years even numbers**
Kay vision test<br>Whisper hearing test<br>KBIT-2<br>CANTAB - MOT<br>CANTAB - PAL<br>Finger nose pointing<br>CANTAB - IED<br>CAMCOG - Delayed incidental memory, verbal fluency and orientation<br>CANTAB - SRT<br>Delayed object memory with gait and measurements in the break for delayed recall<br>Break<br>Tower of London<br>ACTB - finger sequencing<br>NAID - memory for sentences<br>ACTB - virtual computer generated arena<br>NEPSY-II - visuomotor precision<br>ACTB - cats and frogs	Kay vision test<br>Whisper hearing test<br>KBIT-2<br>CANTAB - MOT<br>ACTB - cats and frogs<br>NEPSY-II - visuomotor precision<br>ACTB - virtual computer generated arena<br>NAID - memory for sentences<br>ACTB - finger sequencing<br>Tower of London<br>Break<br>Delayed object memory with gait and measurements in the break for delayed recall<br>CANTAB - SRT<br>CAMCOG - Delayed incidental memory, verbal fluency and orientation<br>CANTAB - IED<br>Finger nose pointing<br>CANTAB - PAL

Box 1. Assessment schedule; the order of assessments is counter-balanced across participants.
