# Supplementary material for: The LonDownS adult cognitive assessment to study cognitive abilities and decline in Down syndrome
Source: Wellcome Open Res. 2016 Nov 15;1:11. [Version 1] doi: 10.12688/wellcomeopenres.9961.1 (PMC5176078; doi:10.12688/wellcomeopenres.9961.1)
Supplement: Peer review history

# Peer review: Referee response for version 1

**DOI:** 10.21956/wellcomeopenres.10736.r18560

**Published:** 2016-12-16

**Authors:** Bessy Benejam

**Affiliations:** 1 Down Medical Centre, Catalan Down Syndrome Foundation, Barcelona, Spain

Down syndrome (DS) is associated with a very high risk of developing Alzheimer’s disease (AD). Diagnosing dementia in individuals with DS can be difficult because of the challenges involved in assessing changes in people with pre-existing cognitive impairment and due to a lack of appropriate instruments. The authors study a very important topic, the search for an appropriate battery of tests suitable for the assessment of cognitive functions of individuals with a wide range of ages and intellectual disability (ID) severities, and for the early detection of cognitive decline associated to the development of AD. Startin and colleagues describe the LonDownS cognitive test battery for adults with Down syndrome (DS), and provide data on baseline cognitive and related informant assessments for two cohorts of participants ( cohort 1: adults aged 36 and over with or without clinical diagnosis of dementia; cohort 2: adults aged 16-35 years).

 The authors found high completion rates across the tasks for individuals without dementia, with many showing low floor and ceiling effects. Results from individuals with dementia suggest lower completion rates (approximately half of the individuals with dementia were unable to undertake any task).

 To my point of view, the manuscript is well written, hypothesis and methods are adequately described, and it is overall well discussed.

 I have some suggestions to improve the manuscript format and some questions that the authors could clarify:

- After reading the title of the manuscript, one expects to find some comparison in task performance between individuals with and without dementia, with the aim to find cognitive tasks able to discriminate well between both groups. I think the manuscript would benefit from the inclusion of a new table with the main outcome measures and results from informant questionnaires for both adults with and without dementia, with its statistical differences.
- The conclusions of the study are well written and described, but I suggest reviewing the “Conclusion” section of the Abstract because it does not explain what the current study found.
- As stated in the “Methods” section (Task administration), **the assessment was completed in one session where possible, approximately 3 hours in duration**. Although this limitation is imposed by the study set up, this might represent a limitation worth mentioning. In my opinion, this duration might be too long for people with intellectual disability, a population that might get tired more easily and has usually difficulties with sustained attention. The validation study conducted by Sinai and colleagues, which found very similar scores for a number of tests (some of them also used by Startin and colleagues) when comparing dementia and no dementia groups of older people with DS, could help to reduce the length of the cognitive test battery.
- The authors recruited 51 adults aged 36 years and over, with a clinical diagnosis of dementia. Of these, 22 (43%) had a diagnosis of AD and 27 (more than 50%) had a diagnosis of dementia of unspecified type. This is a surprising high figure for a population with an ultra-high risk for AD. The manuscript does not describe who made the diagnosis of dementia or how it was made. It could also be interesting to include some information about the stage of the dementia. Which patients were unable to undertake the assessment? Was this related to a more advanced stage of dementia, or was this related to a more severe premorbid intellectual disability?
- The number of tables in the results section might be reduced. Some of them might be included if deemed important as supplementary material. For example, given that there are few differences between males and females, and the findings about the correlation between outcome scores for Cohort 2 are well explained in the results section, I suggest Tables 8 to 11 could be included as supplementary material.

I have read this submission. I believe that I have an appropriate level of expertise to confirm that it is of an acceptable scientific standard.

# Peer review: Referee response for version 1

**DOI:** 10.21956/wellcomeopenres.10736.r17957

**Published:** 2016-12-13

**Authors:** Alessandro Ghezzo

**Affiliations:** 1 Department of Experimental, Diagnostic and Specialty Medicine, Alma Mater Studiorum-University of Bologna, Bologna, Italy

The Authors describe their cognitive test battery to assess neuropsychological abilities in two groups of adults with Down syndrome (DS) (a group of 16-35 years of age and another group aged 36 and over), and related informant measures along with reporting data from their baseline cognitive and informant assessments. The ultimate goal of this study is to put the basis for a longitudinal neuropsychological evaluation of people with DS: The LonDownS Consortium, aimed at determining risk and protective factors for the development of dementia and factors relating to cognitive abilities in people with DS.

 The topic addressed by the authors is of great interest not only for people with DS. In fact, with the increase in life expectancy and the resulting aging of the general population, age-related neurodegenerative disorders, such as neurocognitive impairment, are expected to increase. DS is considered a model of premature aging, and therefore the study of dementia in DS and the evaluation of favouring or protective factors, can bring benefits both for people with DS and for general population. 

1.  A comprehensive assessment
  The Authors performed a very comprehensive neuropsychological evaluation, to assess a range of skills relevant to those brain areas most affected in DS, including general abilities, visuo-spatial and verbal memory, language skills, executive functions and motor coordination abilities.
2. The effort to avoid floor and ceiling effects
  The Authors show not only the results and the related statistical analysis, but also the reasoning why they have chosen that specific test and the scientific background: in my opinion this issue is of great interest, because the neuropsychological evaluation of people with intellectual disability (ID) is a relatively young discipline, and there is a great effort worldwide aimed to identify a test battery specifically suitable for people with ID. 
  In fact, the main problem in the assessment of people with DS, and more generally of people with ID, is that most of the neuropsychological tests were conceived and designed for cognitive evaluation of people without ID. The consequence of this is that often they cannot be used in people with ID, because of the floor and ceiling effects. In both cases, they do not give indications for neuropsychological assessment and for longitudinal studies. The effort of the authors was to identify a set of tests that present minimum percentages of floor and ceiling effects, in order to make them suitable for the neuropsychological assessment of people with DS.
  According to the results this effort has been successful: in this study most of the persons with DS without dementia were able to perform the tests and the percentage of floor and ceiling effect was very low (except for CAMCOG – delayed incidental memory and KBIT-2 age-dependent IQ scores).
3. Administration times
  Another important parameter is given by the administration times. The authors specify that the test administration, most often conducted in the person's home, lasted overall about three hours, including breaks necessary to rest and, wherever possible, physical measurements (height, weight, abdominal/ head/neck circumference, gait, blood pressure, and pulse). In my opinion it is a perfectly acceptable time for such a comprehensive assessment, also in the light of possible follow-up in order to detect early symptoms of cognitive decline.

Some questions and suggestions

 Question 1: Have the Authors performed a correlation between cognitive scores and informant scores?

 Question 2: Have the Authors performed a correlation between age and cognitive/informant scores considering the whole group of persons with DS (16-35 years of age + aged 36 and over)  

 Question 3: Which informant questionnaire can be suggested by the Authors to be used in association with the tests listed in Box1 (Ideal outcome measures to use in future studies)?

 Suggestion: I think that the title of Table 11 is wrong and should be changed in “Correlations between informant scores and age across adults aged 16–35”

I have read this submission. I believe that I have an appropriate level of expertise to confirm that it is of an acceptable scientific standard.
